# Prolonged high-dose intravenous magnesium therapy for severe tetanus in the intensive care unit: a case series

**DOI:** 10.1186/1752-1947-4-100

**Published:** 2010-03-31

**Authors:** Menelaos Karanikolas, Dimitrios Velissaris, Markos Marangos, Vassilios Karamouzos, Fotini Fligou, Kriton S Filos

**Affiliations:** 1 Department of Anaesthesiology and Critical Care Medicine, Patras University Hospital, Rion, 26500, Greece; 2 Department of Internal Medicine, Patras University Hospital, Rion, 26500, Greece

## Abstract

**Introduction:**

Tetanus rarely occurs in developed countries, but it can result in fatal complications including respiratory failure due to generalized muscle spasms. Magnesium infusion has been used to treat spasticity in tetanus, and its effectiveness is supported by several case reports and a recent randomized controlled trial.

**Case presentations:**

Three Caucasian Greek men aged 30, 50 and 77 years old were diagnosed with tetanus and admitted to a general 12-bed intensive care unit in 2006 and 2007 for respiratory failure due to generalized spasticity. Intensive care unit treatment included antibiotics, hydration, enteral nutrition, early tracheostomy and mechanical ventilation. Intravenous magnesium therapy controlled spasticity without the need for additional muscle relaxants. Their medications were continued for up to 26 days, and adjusted as needed to control spasticity. Plasma magnesium levels, which were measured twice a day, remained in the 3 to 4.5 mmol/L range. We did not observe hemodynamic instability, arrhythmias or other complications related to magnesium therapy in these patients. All patients improved, came off mechanical ventilation, and were discharged from the intensive care unit in a stable condition.

**Conclusion:**

In comparison with previous reports, our case series contributes the following meaningful additional information: intravenous magnesium therapy was used on patients already requiring mechanical ventilation and remained effective for up to 26 days (significantly longer than in previous reports) without significant toxicity in two patients. The overall outcome was good in all our patients. However, the optimal dose, optimal duration and maximum safe duration of intravenous magnesium therapy are unknown. Therefore, until more data on the safety and efficacy of magnesium therapy are available, its use should be limited to carefully selected tetanus cases.

## Introduction

Tetanus is a rare and potentially fatal disease caused by tetanospasmin, a *Clostridium tetani *exotoxin. Patients with tetanus manifest generalized muscle rigidity that can cause respiratory failure, thus requiring both admission to an intensive care unit (ICU) and mechanical ventilation. Case reports, uncontrolled case series and a recent randomized controlled trial (RCT) from Vietnam suggest that magnesium (Mg) is an interesting treatment option for tetanus, but concerns regarding Mg therapy risks still exist. We present three patients with tetanus who were admitted to the ICU at Patras University Hospital for respiratory failure. They received prolonged high-dose intravenous Mg therapy and had good outcomes.

## Case presentation

All three patients we describe in this case series were admitted to our hospital's ICU for generalized muscle rigidity and respiratory failure requiring intubation and mechanical ventilation. During their ICU stay all of them received central (subclavian or internal jugular vein) and radial arterial lines. All of them received propofol combined with clonidine for sedation. According to ICU protocol, their hypotension (defined as mean arterial pressure [MAP] <50 mmHg) was treated with continuous intravenous norepinephrine infusion. Our patients' bradycardia, which is defined as having a heart rate of <45/minute, was treated with intravenous atropine and/or continuous isoproterenol infusion. Meanwhile, their tachycardia, which is defined as having a heart rate of >110/min, was treated with continuous esmolol infusion. All our patients received low molecular weight heparin for deep venous thrombosis prophylaxis and enteral nutrition through an orogastric tube. Their urine output was maintained above 0.5 mL/kg/hour by using appropriate intravenous fluid administration.

The effectiveness of Mg therapy was evaluated with daily wake-up tests in order to assess muscle rigidity and the ability of our patients to breathe spontaneously with pressure support while awake. When the wake-up test indicated insufficient rigidity control, their Mg dose was increased by 10% to 15%. On the other hand, when no rigidity was noted the dose was reduced by 10% to 25%. Data collection was retrospective.

## Case report 1

A 50-year-old Caucasian Greek man with a history of excess alcohol use, hepatitis C virus (HCV) infection and intravenous drug use was admitted to the ICU for generalized spasms, opisthotonus and severe respiratory distress. He had received a tetanus vaccination in childhood and a booster tetanus vaccine during military service in his 20s, but had not received any tetanus vaccination in 20 years. Upon arrival at the ICU he required intubation and positive pressure ventilation. Initial antibiotic therapy consisted of meropenem, vancomycin and metronidazole. Colistin and gentamicin were added seven days later for *Enterobacter cloacae *pneumonia with positive blood cultures.

Because of hypotension, he received continuous intavenous norepinephrine infusion for five days during an episode of sepsis. Because his condition was critical, and in our judgment he was unlikely to come off the ventilator quickly, an elective percutaneous tracheostomy was performed on his third day in the ICU. His muscle rigidity improved within a few hours after we started him on Mg infusion. However, as his muscle rigidity on daily wake-up tests persisted, his intravenous Mg infusion continued for 26 days, with a total Mg dose of 337 g. His plasma Mg levels were measured daily and remained in the 3 to 4 mmol/L range. Our patient gradually improved, was weaned off the ventilator, and was discharged from the ICU in a stable condition after 30 days.

## Case report 2

A 77-year-old Caucasian Greek man without any significant medical history was admitted to the ICU for respiratory failure due to generalized spasticity. A week earlier he had had a right foot injury that had resulted in a small, tender, erythematous, palpable mass. He did not have any written immunization records, but he was certain that he had not received any tetanus vaccine in over 20 years.

Upon his arrival at the ICU he was sedated, intubated and supported with positive pressure ventilation. His muscle rigidity was initially treated with analgesia, sedation and intermittent intravenous cisatracurium boluses. His antibiotic therapy included ceftriaxone and metronidazole. We performed a percutaneous tracheostomy on our patient on his seventh day in the ICU. As his muscle rigidity persisted, we initiated him on continuous intravenous Mg infusion on day 8 in an attempt to reduce his need for non-depolarizing muscle relaxants. His rigidity improved significantly after he was started on Mg infusion, and within a few hours he no longer required non-depolarizing muscle relaxants. His plasma Mg levels were measured daily. His spasticity was well-controlled, with his Mg plasma levels maintained within the 4 to 4.5 mmol/L range. Our patient came off the ventilator on day 14 and his Mg infusion was stopped on day 16. He was discharged from the ICU in a stable condition on day 22.

## Case report 3

A 30-year-old Caucasian Greek man was admitted to the ICU with a diagnosis of tetanus manifesting as generalized spasms, trismus and dysphagia. He had apparently self-administered heroin with a contaminated needle three days before he became ill. He had received a tetanus vaccination during childhood, but had not had a booster tetanus vaccine for at least 10 years. On ICU admission his hemodynamic variables were stable. He was started immediately on intravenous Mg therapy and his rigidity clearly improved after 3 to 4 hours. Because of persistent rigidity in his daily wake-up tests, however, his Mg therapy continued for 26 days. His antibiotic therapy included meropenem, vancomycin and metronidazole. His airway was secured with percutaneous tracheostomy on day 5. After a prolonged ICU stay without major complications, his intravenous Mg therapy was discontinued on day 26. He was weaned off the ventilator on day 28 and was discharged from the ICU in a stable condition on day 32.

Demographic, therapy and outcome data are presented in Table 1. Autonomic nervous system instability was not a problem, as all patients (except for patient 1 during a 5-day episode of sepsis) maintained cardiovascular stability. Intravenous Mg therapy resulted in excellent muscle spasm control for our patients within hours, without the use of any additional muscle relaxants. However, due to the persistence of painful muscle rigidity during their daily wake-up tests, intravenous Mg therapy needed to be continued for a long period (26 days) in patients 1 and 3. Our patients tolerated intravenous Mg therapy well without any significant adverse effects, and they were discharged from the ICU in a stable condition.

**Table 1:** Demography, treatment and outcome data

Case	Age/Gender	PMH	Hemodynamic instability	Inotropic agents	Total Mg dose (g)	Mg therapy (days)	Additional sedatives/Relaxants	Ventilation (days)	ICU stay (days)	Outcome
1	50/M	Alcohol, IV drugs, Tobacco, HCV	HR >110/min, MAP <50 mmHg on ICU admission	NE 5 days	337	26	Propofol 200-400 mg/hour Clonidine 2100 μg/day	22	30	Good
2	77/M	HTN, Tobacco	None	None	277	7	Propofol 200-300 mg/hour Cisatracurium PRN, Fentanyl PRN for 1 week, until Mg started	14	22	Good
3	30/M	IV drugs, Tobacco, HCV	None	None	758	26	Propofol 300-500 mg/hour Clonidine 3000 μg/day	28	32	Good

HCV: hepatitis c virus; HR: heart rate; HTN: hypertension; ICU: intensive care unit; IV: intravenous; M: male; Mg: magnesium; MAP: mean arterial pressure; NE: norepinephrine, PMH: past medical history; PRN: Pro re nata (As Needed).

## Discussion

Tetanus is a disease of the nervous system and can present in one of four forms: generalized, localized, cephalic and neonatal [1]. In adults, the generalized form, manifesting as skeletal muscle rigidity and convulsive muscle spasms, is the most severe. Urbanization, agriculture mechanization and socioeconomic factors, including poverty, poor hygiene and lack of health services, significantly influence tetanus incidence. Although the number of tetanus cases has been declining every year in the last two decades due to improved vaccination practices, there are still up to 500,000 cases of tetanus recorded yearly worldwide.

Tetanus mortality is as high as 45% [2-4]. A total of 75% of deaths occur within the first week, primarily from pulmonary infection, aspiration or pulmonary embolism. Up to 163,000 deaths were attributed to tetanus in 2004 [5].

Tetanus incidence is markedly low in developed countries. Between 1972 and 2001, only 1842 tetanus cases were reported in the United States [3]. The annual incidence per million decreased from 0.39 in 1972 to 1976 to 0.16 in 1997 to 2001, while case fatality rate (CFR) decreased from 45% to 16% [3]. Among the reported 932 patients recorded, 644 (69%) were unvaccinated. Meanwhile, CFR was recorded at 28%. Tetanus incidence and mortality are highest in those aged over 60 (with an incidence of 0.78 per million, CFR 40%). Diabetes is strongly associated with the risk of fatal tetanus (age-adjusted relative risk = 1.9; 95% confidence interval [CI] at 1.4 to 2.6). Approximately 50% of tetanus cases in the USA occur after injuries [1], but intravenous drug use is becoming increasingly significant. Injection drug users accounted for 12% of tetanus cases in 1992 to 2001, a three-fold increase compared with the previous decade [3].

After *Clostridium tetani *spores enter human tissues, they convert to vegetative forms, multiply (often without signs of local inflammation or infection), and release tetanospasmin [1]. Premonitory symptoms are non-specific and include restlessness, irritability, headache, jaw pain and stiffness, back or abdominal pain, and difficulty in swallowing. Trismus (lock jaw) is the most common symptom, but tachycardia, low-grade fever and profuse sweating are also common. Patients with tetanus are almost invariably conscious and alert when they seek medical attention. As there are no laboratory findings specific to tetanus [6], diagnosis is based on history and clinical symptoms. As more muscles become involved, generalized rigidity can result in respiratory complications, including hypoxia and atelectasis. Other complications include deep venous thrombosis, pulmonary embolism and cardiovascular instability (hypertension, tachycardia, arrhythmias and severe vasoconstriction). The treatment for tetanus usually requires hospitalization, placement in a quiet room and observation for developing complications. Care of patients with tetanus should include monitoring of vital signs, aspiration of nasopharyngeal secretions, maintenance of fluid and electrolyte balance, and treatment of rigidity. Early tracheostomy should be considered because it protects against suffocation from laryngospasm, reduces aspiration risk, and facilitates mechanical ventilation [7-9].

The main pathophysiological disturbances in tetanus are caused by tetanospasmin, a neurotoxin produced by *Clostridium tetani*. Tetanospasmin enters the nervous system at the neuromuscular junction, migrates towards the central nervous system by retrograde axonal transport, and is also carried by lymphocytes. Tetanospasmin binds at the presynaptic nerve ending of the neuronal membrane and blocks inhibitory amino acid (gamma aminobutyric acid [GABA] and glycine) release. Consequently, the absence of inhibitory GABA and glycine impulses results in spasms, seizures and sympathetic overactivity [1].

Magnesium is a presynaptic neuromuscular blocker with vasodilator, catecholamine release blocking and anticonvulsant properties, all of which are desirable for spasticity and autonomic dysfunction control in tetanus. The therapeutic use of Mg in tetanus may cause temporary muscle weakness or paralysis. It may also lead to reduced sympathetic activity resulting in vasodilation, blood pressure reduction and lowering of heart rate [10]. Although all these effects of Mg therapy are desirable, problems from excessive weakness and hypotension have been described [11].

Clinical data, including case reports [10] and uncontrolled case series [12,13], suggest that Mg therapy is effective in managing tetanus. A prospective observational study from Sri Lanka [11] and a recent large RCT from Vietnam [14] also support the safety and efficacy of Mg therapy in cases of severe tetanus. Mg reduces the need for medications to control muscle rigidity and cardiovascular instability [10,13,14] but does not reduce mortality and the need for mechanical ventilation [14]. However, as Mg therapy can result in serious adverse effects, including muscle weakness, paralysis and hypotension, additional data are needed before Mg is accepted as the first-line therapy for tetanus [15-17].

In our report we describe three patients with tetanus and respiratory failure who required prolonged mechanical ventilation and ICU care. All three patients received prolonged continuous high-dose intravenous Mg therapy, had early percutaneous tracheostomy, and were successfully weaned off mechanical ventilation when their spasticity improved. Except for a single sepsis episode in one of our patients, they did not exhibit hemodynamic instability during treatment. All our patients left the ICU in a stable condition, and did not need further treatment for spasticity.

Most reports on intravenous Mg therapy for tetanus come from developing countries such as Sri Lanka [11] and Vietnam [14]. In most published cases, intravenous Mg therapy was used in an attempt to avoid mechanical ventilation in the context of scarce ICU resources. Our case series may be the only report on the use of intravenous Mg therapy in the European Union, where tetanus is rare. In an attempt to describe the use of intravenous Mg in patients with tetanus already requiring mechanical ventilation, our report may have included sicker patients than those described in other publications. Our reason for using Mg therapy was to avoid the use of non-depolarizing muscle relaxants and not to avoid mechanical ventilation. In addition, two of the three patients we described in this report received intravenous Mg therapy for a very long period (26 days), which is significantly longer than described in previous reports.

The observation that high-dose intravenous Mg therapy, if carefully titrated and monitored, can continue for a long time without obvious adverse effects or major organ toxicity is, in our opinion, the most interesting finding in our report. Therefore, we believe that this report is a meaningful addition to the literature on Mg therapy for tetanus-related spasticity in patients requiring prolonged mechanical ventilation in the ICU.

Our experience suggests that prolonged high-dose intravenous Mg infusion therapy is effective in managing tetanus and can be implemented without major toxicity. However, Mg therapy can be associated with serious adverse effects, and our data are insufficient to confirm the safety of this therapy. Until more data from RCTs are available, we believe that high-dose intravenous Mg therapy is a promising treatment option. It should, however, be reserved for selected patients who have intractable spasticity despite adequate sedation and analgesia and who may otherwise require prolonged therapy with non-depolarizing muscle relaxants.

## Conclusion

As tetanus is becoming rare due to widespread vaccination, experience in treating severe tetanus in developed countries is limited. The effectiveness of intravenous Mg therapy for spasticity in tetanus is supported by case reports, uncontrolled trials and a recent RCT. We present three patients with severe tetanus complicated by respiratory failure and who, as such, require mechanical ventilation. All three patients required ICU treatment and prolonged intravenous Mg infusion at unusually high doses. Mg toxicity never became a problem, and all three patients improved and were discharged from the ICU in a stable condition. This report provides additional evidence supporting the use of Mg therapy in severe tetanus. However, as Mg therapy can have significant adverse effects, additional data from large RCTs that confirm its efficacy and safety are needed before Mg therapy can be accepted as the first-line therapy for tetanus.

## Abbreviations

CFR: case fatality rate; CNS: central nervous system; DVT: deep venous thrombosis; HCV: hepatitis C virus; ICU: intensive care unit; IV: intravenous; NE: norepinephrine; RCT: randomized controlled trial.

## Consent

Written informed consent was obtained from our patients for publication of this case series and any accompanying images. A copy of the written consent is available for review by the Editor-in-Chief of this journal.

## Competing interests

The authors declare that they have no competing interests.

## Authors' contributions

MK introduced Mg therapy for tetanus in the ICU and wrote the manuscript. DV provided patient care, collected data, and edited the manuscript. MM participated in patient care, directed antibiotic therapy, and edited the manuscript. VK collected data and helped with manuscript editing and submission. FF provided patient care, collected data and edited the manuscript. KF directed patient care and edited the manuscript. All authors read and approved the final manuscript.
